# Impaired right ventricular contractile function in childhood obesity and its association with right and left ventricular changes: a cine DENSE cardiac magnetic resonance study

**DOI:** 10.1186/s12968-017-0363-5

**Published:** 2017-06-28

**Authors:** Linyuan Jing, Arichanah Pulenthiran, Christopher D. Nevius, Abba Mejia-Spiegeler, Jonathan D. Suever, Gregory J. Wehner, H. Lester Kirchner, Christopher M. Haggerty, Brandon K. Fornwalt

**Affiliations:** 1 Department of Imaging Science and Innovation, Geisinger Health System, 100 North Academy Avenue, Danville, 17822-4400 PA USA; 2 Biomedical and Translational Informatics Institute, Geisinger Health System, Danville, PA USA; 3 Department of Biomedical Engineering, University of Kentucky, Lexington, KY USA; 4 Department of Radiology, Geisinger Health System, Danville, PA USA

**Keywords:** DENSE, Right ventricle, Strain, Pediatric obesity

## Abstract

**Background:**

Pediatric obesity is a growing public health problem, which is associated with increased risk of cardiovascular disease and premature death. Left ventricular (LV) remodeling (increased myocardial mass and thickness) and contractile dysfunction (impaired longitudinal strain) have been documented in obese children, but little attention has been paid to the right ventricle (RV). We hypothesized that obese/overweight children would have evidence of RV remodeling and contractile dysfunction.

**Methods:**

One hundred and three children, ages 8–18 years, were prospectively recruited and underwent cardiovascular magnetic resonance (CMR), including both standard cine imaging and displacement encoding with stimulated echoes (DENSE) imaging, which allowed for quantification of RV geometry and function/mechanics. RV free wall longitudinal strain was quantified from the end-systolic four-chamber DENSE image. Linear regression was used to quantify correlations of RV strain with LV strain and measurements of body composition (adjusted for sex and height). Analysis of variance was used to study the relationship between RV strain and LV remodeling types (concentric remodeling, eccentric/concentric hypertrophy).

**Results:**

The RV was sufficiently visualized with DENSE in 70 (68%) subjects, comprising 36 healthy weight (13.6 ± 2.7 years) and 34 (12.1 ± 2.9 years) obese/overweight children. Obese/overweight children had a 22% larger RV mass index (8.2 ± 0.9 vs 6.7 ± 1.1 g/m^2.7^, *p* < 0.001) compared to healthy controls. RV free wall longitudinal strain was impaired in obese/overweight children (−16 ± 4% vs −19 ± 5%, *p* = 0.02). Ten (14%) out of 70 children had LV concentric hypertrophy, and these children had the most impaired RV longitudinal strain compared to those with normal LV geometry (−13 ± 4% vs −19 ± 5%, *p* = 0.002). RV longitudinal strain was correlated with LV longitudinal strain (*r* = 0.34, *p* = 0.004), systolic blood pressure (*r* = 0.33, *p* = 0.006), as well as BMI z-score (*r* = 0.28, *p* = 0.02), waist (*r* = 0.31, *p* = 0.01), hip (*r* = 0.40, *p* = 0.004) and abdominal (*r* = 0.38, *p* = 0.002) circumference, height and sex adjusted.

**Conclusions:**

Obese/overweight children have evidence of RV remodeling (increased RV mass) and RV contractile dysfunction (impaired free wall longitudinal strain). Moreover, RV longitudinal strain correlates with LV longitudinal strain, and children with LV concentric hypertrophy show the most impaired RV function. These results suggest there may be a common mechanism underlying both remodeling and dysfunction of the left and right ventricles in obese/overweight children.

**Electronic supplementary material:**

The online version of this article (doi:10.1186/s12968-017-0363-5) contains supplementary material, which is available to authorized users.

## Background

Childhood obesity is a rapidly growing public health problem. Recent studies in the United States show that 17% of children and adolescents (2–19 years) are obese, while 5.8% are severely obese [1]. A longitudinal study which followed over two million adolescents into adulthood showed that pediatric obesity is linked to increased risk of mortality in adulthood [2]. Although the exact mechanisms are not well understood, evidence suggests that this premature mortality is likely attributed to early onset of cardiovascular diseases [3].

Prior research has focused primarily on changes in the structure and function of the left ventricle (LV) in obese/overweight children using echocardiography or cardiovascular magnetic resonance (CMR). Major findings include: LV remodeling, evidenced by increased LV myocardial mass and wall thickness [4–6]; contractile dysfunction, measured by impaired LV longitudinal and circumferential strain [6–8]; and diastolic dysfunction, indicated by reduced early (E) and late (A) relaxation velocity as well as decreased E/A ratio [4, 5]. Moreover, approximately 25% of obese children have LV concentric hypertrophy [9], the type of remodeling that is most closely related to early mortality in adults [10]. A recent CMR study showed that obese children with LV concentric hypertrophy demonstrate the most impaired LV longitudinal and circumferential strains, despite normal ejection fraction [6].

Despite mounting evidence of LV remodeling and dysfunction, little is known about the right heart in obese children. There is growing appreciation that right ventricular (RV) dysfunction is related to adverse outcomes [11–13]. RV remodeling (larger RV mass and volumes) and impaired systolic and diastolic function have been documented in overweight and obese adults [14–17]. Moreover, there is evidence of potential links between LV and RV function in obese adults [14]. Thus, obese children with LV concentric hypertrophy and impaired LV strain/function may also suffer from RV remodeling and impaired RV function. Information on RV geometry and function could facilitate the identification of obese children who are at high risk of adverse outcomes, as well as provide new insights into the etiology underlying cardiac dysfunction in the setting of pediatric obesity.

Only a few studies have investigated obesity-related changes in RV geometry and function in children, but the findings are conflicting [4, 5, 18–21]: both no change and a decrease in RV systolic function were reported. These conflicting reports may result from the fact that all previous studies have utilized echocardiography (tissue Doppler imaging) to quantify RV function. This methodology suffers from poor image quality and high angle dependency of the imaging plane, especially in the RV [22]. These limitations preclude direct and comprehensive characterization of RV structure and function with echocardiography. Magnetic resonance imaging has excellent image quality and is the gold standard for reproducibly imaging the RV [23]. Moreover, cine displacement encoding with stimulated echoes (DENSE), an advanced CMR technique, encodes displacement of myocardial tissue into CMR phase images, providing adequate resolution to quantify displacement and strain of the RV [24]. To our knowledge, no study has investigated changes in RV structure and function in obese children using CMR. We hypothesized that obese children would have enlarged RV mass as well as impaired contractile function (strain), and that these changes would be associated with LV remodeling and function.

## Methods

### Study population

Children ages 8–18 years were prospectively recruited from University of Kentucky (the High BMI Diagnostic Clinic, and the Center for Clinical and Translational Science volunteer database) and Geisinger Medical Center. Children were categorized based on their body mass index (BMI) percentiles defined by the United States Centers for Disease Control and Prevention (CDC) growth charts [25]: obese (BMI ≥95^th^ percentile), overweight (BMI 85^th^–95^th^ percentile) and healthy weight (BMI 5^th^–85^th^ percentile). Exclusion criteria included diabetes, diagnosed hypertension, history of heart disease, contraindications for CMR, or a waist circumference >125 cm due to the circumference limitation of the bore of the CMR scanner. Children with conditions that could potentially alter right heart function (e.g. obstructive sleep apnea, pulmonary hypertension or lung disease) were excluded. A subgroup (two-thirds) of the subjects were included in a previous study focused on LV remodeling and function (strain) [6].

### Clinical assessment

At the time of the CMR scan, height and weight, each averaged from two readings, were measured, and BMI (weight/height^2^ in kg/m^2^) percentiles were determined. Measurements of the waist, abdominal, and hip circumferences were taken twice with a tailor’s scale, and the average values were reported. After being seated for at least 10 min, an appropriately sized cuff was used to measure resting blood pressure by auscultation three times, 5 min apart. The average of the last two readings was reported. All children had a normal 12-lead electrocardiogram (ECG).

### CMR imaging

All subjects underwent CMR on a 3 T system (Tim Trio, Siemens Healthcare, Erlangen, Germany) using 6-element chest and 24-element spine coils. All CMRs were conducted solely for research purposes and not for clinical indications. Standard ECG-gated balanced steady-state free-precession (SSFP) images in two- and four-chamber views were acquired during 10–15 s breath-holds. Depending on the size of the heart, a stack of 7–11 short-axis SSFP images spanning both ventricles were acquired for assessment of cardiac geometry. Acquisition parameters were: repetition time (TR) = 3.16–3.37 ms, echo time (TE) = 1.3–1.5 ms, field of view (FOV) = [292–400] × [340–400] mm^2^, image matrix = [208–256] × 256, flip angle = 50°, temporal resolution = 16.4–49.9 ms, slice thickness = 8 mm, slice gap = 0–3.7 mm.

Spiral cine DENSE imaging was performed in the two-chamber and four-chamber long-axis views to quantify LV and RV longitudinal strain. A respiratory navigator with an acceptance window of ±3 mm was used to maintain consistent breath-hold position. The acquisition parameters for DENSE images were: 6 or 18 spiral interleaves, in-plane simple displacement encoding (k_e_ = 0.1 cycles/mm), TE/TR = 1.08/17 ms, flip angle = variable 20°, voxel size = 2.8 × 2.8 × 8 or 1.6 × 1.6 × 8 mm^3^, FOV = 360 × 360 or 340 × 340 mm^2^, image matrix = 128 × 128 or 214 × 214, temporal resolution = 34 ms (view sharing was used to achieve 17 ms between reconstructed temporal frames). CSPAMM was used for echo suppression.

### Image analysis

Endocardial and epicardial boundaries of the RV were manually delineated on end-diastolic and end-systolic frames on all short-axis SSFP slices covering the whole ventricle. Simpson’s rule was used to calculate RV end-diastolic (EDV) and end-systolic (ESV) volumes. RV stroke volume (SV = EDV - ESV) and ejection fraction (SV/EDVx100%) were also derived. RV myocardial mass was calculated from the epicardial and endocardial contours on the end-diastolic frame, assuming a myocardial density of 1.05 g/mL, and indexed to height (meters^2.7^).

**Fig. 1:**
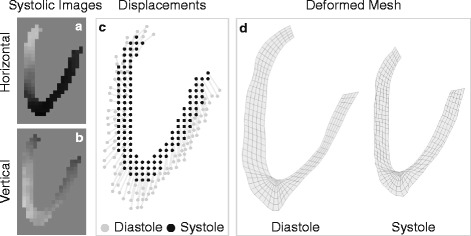
Right ventricular (*RV*) strain analysis. End-systolic phase images encoding the *horizontal* (**a**) and vertical (**b**) displacements were acquired using 2D spiral cine DENSE. The displacements for each pixel in the end-systolic image (*black dots* in **c**) relative to end-diastole (*grey dots* in **c**) were extracted directly from the phase images. A mesh of the RV myocardium was deformed from diastole to systole using these displacements (**d**), and strains were computed for each mesh element

RV free wall longitudinal strain was quantified from the four-chamber DENSE acquisition (Fig. 1). End-systolic phase images encoding the horizontal (Fig. 1a) and vertical (Fig. 1b) displacements were acquired using 2D spiral cine DENSE. The RV free wall and the inter-ventricular septum were segmented using *DENSEanalysis* [26]. DENSE post-processing was customized [27] to remove temporal smoothing and instantaneously quantify strain using only the end-systolic frame, as determined visually based on the minimum chamber area. Specifically, the displacements for each pixel in the systolic image (black dots in Fig. 1c) relative to end-diastole (grey dots in Fig. 1c) were extracted directly from the phase images. Using these displacements, a mesh of the RV myocardium was deformed from diastole to systole (Fig. 1d) and strains were computed for each mesh element. Only RV free wall elements were retained for the analysis. Notably, the analysis of a single time phase in this way was the original approach used for DENSE imaging, prior to the development of the cine version of DENSE [28].

Standard DENSE post-processing was performed to compute LV longitudinal strain in order to study its relationship with RV strain. Analysis included manual segmentation of the LV myocardium, phase unwrapping, tissue tracking throughout the cardiac cycle to derive displacements of the myocardial tissue, and strain calculation as previously described [29, 30]. LV peak longitudinal strain was averaged from the two- and four-chamber views.

To investigate the relationship between RV function/strain and LV remodeling, epicardial and endocardial boundaries of the LV were manually drawn on end-diastolic and end-systolic frames of the SSFP slices. These contours were then used to reconstruct 3D endocardial and epicardial surfaces using a custom algorithm written in MATLAB (The Mathworks, Natick, MA) as previously described [6]. LV volumes, LV myocardial mass (assuming a myocardial density of 1.05 g/mL), and ejection fraction were then calculated from the 3D models. LV myocardial mass was indexed to height (LVMI, grams/meters^2.7^) [31]. Cardiac remodeling types (normal geometry, concentric remodeling, eccentric hypertrophy, concentric hypertrophy) were determined based on previously defined cutoff values for mass/volume ratio (0.69) and LVMI (27.52 g/m^2.7^) in a healthy cohort [6].

### Reproducibility

To quantify inter-observer reproducibility of RV longitudinal strain in all subjects, the end-systolic frame of the DENSE images was independently determined, segmented and analyzed by two different investigators. Bland-Altman limits of agreement and bias were computed [32].

### Statistics

Continuous variables from obese/overweight and healthy groups were compared with a 2-sample student’s *t*-test and presented as mean ± standard deviation (SD). Fisher’s exact test was used to compare the sex distribution between groups. Analysis of covariance (ANCOVA) was used to estimate the differences in cardiac remodeling and function between groups, while accounting for age.

Analysis of Variance (ANOVA) was used to test for a difference in RV strains among different LV remodeling types. Due to the small observed sample sizes, the concentric remodeling and eccentric hypertrophy groups were combined. The concentric hypertrophy group and the combined concentric remodeling/eccentric hypertrophy group were both compared to the normal geometry group using Dunnett’s multiple comparison procedure. Pearson’s correlation coefficients were used to investigate relationships between LV and RV structural and functional measurements.

A modified coefficient of variation (CoV), which compares the variability of a given variable × relative to its mean, was used to quantify inter-observer reproducibility for RV longitudinal strain [33]. A CoV within 20% was considered reproducible. Given measurements from two observers (Ob1 and Ob2), the CoV was calculated as follows:

$$ C o V = \frac{{\displaystyle {\sum}_{i=1}^N}\left[ St. Dev.{\left({X}_{Ob1},\ {X}_{Ob2}\right)}_i\right]}{\left|{\displaystyle {\sum}_{i=1}^N}\left[{\left(\left({X}_{Ob1} + {X}_{Ob2}\right)/2\right)}_i\right]\right|} $$

Multivariable linear regression was used to investigate the relationship between body composition (BMI z-score, waist, hip, abdominal circumference, and waist/hip ratio) and RV remodeling and function. Height and sex were controlled in the model to account for somatic growth and sex differences. Statistical significance was defined as p ≤ 0.05. All statistical analyses were performed in R [34] (Version 3.3.1 with packages multcomp [35] and car [36]).

## Results

### Demographics and clinical assessment of the study population

A total of 103 subjects were prospectively recruited and underwent CMR. Of those, two subjects did not complete the CMR study. In 31 (30%) subjects, the RV was either insufficiently visualized or the phase images were not able to be unwrapped properly during post-processing due to poor image quality. The remaining 70 (68%) subjects, including 36 healthy weight and 34 overweight/obese children, were included in subsequent data analysis. Characteristics of these subjects are summarized in Table 1.

**Table 1:** Demographics and Clinical Parameters (mean ± SD) of the Study Population

	Obese/Overweight *n* = 34	Healthy *n* = 36	*p*
*Age (years)*	12.1 ± 2.9	13.6 ± 2.7	0.03
*Female (%)*	56	47	0.49
*Weight (kg)*	71 ± 23	50 ± 14	<0.001
*Height (cm)*	155 ± 13	160 ± 17	0.23
*Body Mass Index (kg/m* ^*2*^ *)*	29 ± 6	19 ± 2	<0.001
*Body Mass Index Percentile*	96 ± 4	48 ± 23	<0.001
*Body Mass Index z-score*	2.0 ± 0.4	−0.1 ± 0.7	<0.001
*Heart rate (beats/min)*	72 ± 9	70 ± 8	0.09
*Systolic blood pressure (mmHg)*	116 ± 13	109 ± 8	0.02
*Diastolic blood pressure (mmHg)*	73 ± 6	71 ± 6	0.32
*Mean arterial pressure (mmHg)*	87 ± 8	84 ± 6	0.07
*Abdominal Circumference (cm)*	94 ± 16	72 ± 7	<0.001
*Waist Circumference (cm)*	85 ± 14	67 ± 6	<0.001
*Hip Circumference (cm)*	101 ± 16	84 ± 10	<0.001
*Waist/Hip Ratio*	0.85 ± 0.09	0.80 ± 0.07	0.01

Compared to healthy weight children, systolic blood pressure was slightly elevated in the obese/overweight group. In addition, waist, hip, and abdominal circumferences were substantially larger (by 27, 20 and 31%, respectively) in obese/overweight children (all *p* < 0.001). The waist/hip ratio was also higher in obese/overweight children.

We further investigated the characteristics of the 30% of subjects who were excluded from the data analysis. No significant differences were identified in demographic parameters between the excluded subjects and those included for subsequent analysis [see Additional file 1: Table S1]. In addition, the weight group distribution was similar between the two groups: 18 (58%) of the 31 excluded subjects were obese/overweight compared to 34 (49%) out of 70 in the included group, *p* = 0.4.

### Right ventricular geometry and function

**Fig. 2:**
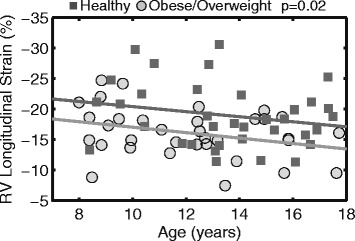
Obese children across all ages have reduced longitudinal strain in the free wall of the *RV*. There was no difference between the slopes of the two *lines* while the intercepts were significantly different (*p* = 0.02), suggesting a difference in *RV longitudinal strain* independent of age between the *healthy* and *obese/overweight* groups

After adjusting for age, obese/overweight children had a 22% larger RV mass index (8.2 ± 0.9 vs 6.7 ± 1.1 g/m^2.7^, *p* < 0.001), while RV volumes were comparable to healthy controls (Table 2). There was no difference in RV ejection fraction between the two groups. However, RV free wall longitudinal strain was impaired in obese/overweight children compared to healthy weight controls (−16 ± 4% vs −19 ± 5%, *p* = 0.02, Fig. 2).

**Table 2:** Cardiac Geometry and Function (mean ± SD)

	Obese/Overweight *n* = 34	Healthy^a^ *n* = 36	*p, age adjusted*
*LV geometry and function*			
*LV mass index (g/m* ^*2.7*^ *)*	27 ± 4	22 ± 3	**<0.001**
*LV end diastolic volume (mL)*	135 ± 31	133 ± 41	0.85
*LV mass/volume ratio*	0.68 ± 0.10	0.60 ± 0.06	**<0.001**
*LV ejection fraction (%)*	62 ± 5	62 ± 4	0.99
*RV geometry and function*			
*RV end diastolic volume (mL)*	149 ± 38	147 ± 46	0.80
*RV end systolic volume (mL)*	60 ± 17	59 ± 23	0.84
*RV stroke volume (mL)*	89 ± 23	88 ± 25	0.80
*RV mass (g)*	27 ± 6	24 ± 7	**0.01**
*RV mass index (g/m* ^*2.7*^ *)*	8.2 ± 0.9	6.7 ± 1.1	**<0.001**
*RV ejection fraction (%)*	60 ± 5	61 ± 5	0.68
*Strain*			
*LV longitudinal strain (%)*	−14 ± 2	−15 ± 2	**0.02**
*RV free wall longitudinal strain (%)*	−16 ± 4	−19 ± 5	**0.02**

*Abbreviations*: *LV* left ventricular, *RV* right ventricular

^a^One of the 36 healthy weight subjects did not complete cine SSFP imaging and was therefore not included in any of the measures of cardiac geometry or ejection fraction

### Association between LV and RV remodeling and function

**Fig. 3:**
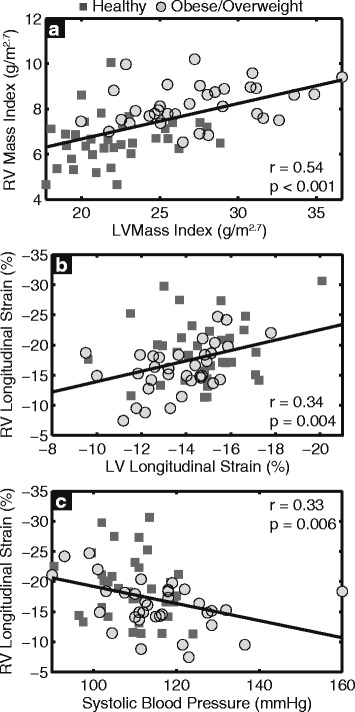
Right ventricular (*RV*) *mass index* correlated with left ventricular (*LV*) *mass index* (**a**); RV free wall *longitudinal strain* was significantly associated with LV *longitudinal strain* (**b**) and systolic blood pressure (**c**)

**Fig. 4:**
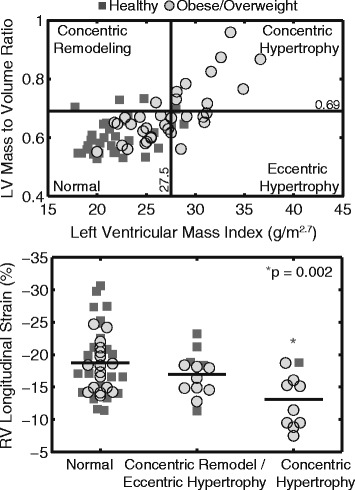
Children with concentric LV hypertrophy (*top*) had the most impaired *longitudinal strain* in the RV free wall (*bottom*). *The reported *p*-value (0.002) is for the comparison between the *concentric hypertrophy* and *normal geometry* group

Consistent with previous studies [4–6], obese/overweight children had a greater LVMI and a higher mass/volume ratio compared to healthy controls (Table 2) after adjusting for age. Furthermore, LVMI correlated with RV mass index (Fig. 3a). Based on cutoff values defined in a previous study [6], ten (14%) subjects had concentric LV hypertrophy, thirteen (19%) subjects had eccentric LV hypertrophy or concentric LV remodeling, and forty-six (66%) had normal LV geometry (Fig. 4). RV free wall longitudinal strain differed among these remodeling groups (*p* = 0.01, Fig. 4), as children with concentric LV hypertrophy had significantly impaired RV strain compared to those with normal LV geometry (−13 ± 4% vs −19 ± 5%, *p* = 0.002). There was no significant difference between the combined concentric remodeling/eccentric hypertrophy group and the normal geometry group (−17 ± 3% vs −19 ± 5%, *p* = 0.46).

In addition, obese/overweight children had impaired LV longitudinal strain (−14 ± 2% vs −15 ± 2%, *p* = 0.02, Table 2) compared to healthy controls, and the LV longitudinal strain correlated with RV longitudinal strain (Fig. 3b). Finally, systolic blood pressure also correlated with RV longitudinal strain (Fig. 3c).

### Correlation between RV function and body composition

**Fig. 5:**
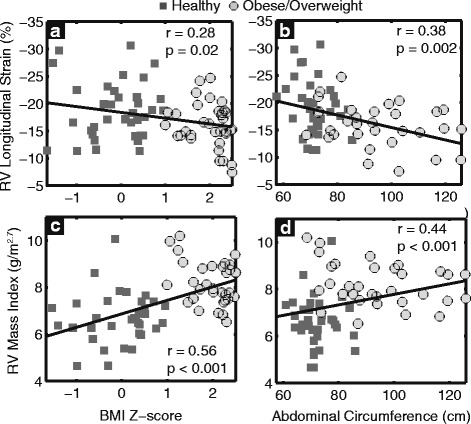
RV free wall *longitudinal strain* and *RV mass index* moderately correlated with *BMI z-score* (**a** and **c**) and *abdominal circumference* (**b** and **d**)

Associations of RV function and strain with body composition measurements are summarized in Table 3. Sex and height were included in the multivariate linear regression model to account for somatic growth. Both RV mass index and RV free wall longitudinal strain moderately correlated with most measurements of body composition except waist/hip ratio (Fig. 5), and the correlations were stronger with RV mass index. RV mass index correlated most strongly with BMI z-score (*r* = 0.56, *p* < 0.001), while RV strain was more strongly correlated with abdominal (*r* = 0.38, *p* = 0.002) and hip (*r* = 0.40, *p* = 0.004) circumferences.

**Table 3:** Correlation between RV Function and Body Composition after Adjusting for Sex and Height

	RV Longitudinal Strain		RV Mass Index	
	*r*	*p*	*r*	*p*
BMI z-score^a^	0.28	**0.02**	0.56	**<0.001**
Waist circumference	0.31	**0.01**	0.44	**<0.001**
Hip circumference	0.40	**0.004**	0.46	**<0.001**
Abdominal circumference	0.38	**0.002**	0.44	**<0.001**
Waist/Hip Ratio	0.07	0.61	0.26	0.052

Multivariate linear regression was used for each pair of predictor (body composition) and outcome (function) variables with adjustment for sex and height

^a^BMI z-score was only adjusted for sex

### Reproducibility

**Fig. 6:**
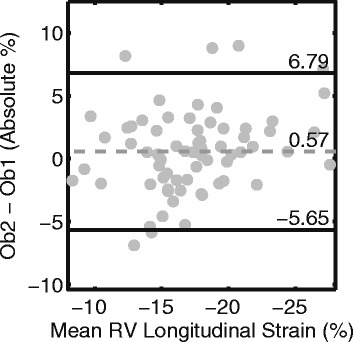
Bland-Altman *plot* shows good reproducibility for *right* ventricular free wall *longitudinal strain* between two observers. *Ob2*: observer 2; *Ob1*: observer 1

The CoV for inter-observer reproducibility of RV longitudinal strain was 10%. This coefficient corresponds with a bias of 0.57 and 95% limits of agreement of [−5.65, 6.79] % (Fig. 6).

## Discussion

We imaged 70 children (36 healthy weight and 34 obese/overweight) with CMR to quantify changes in RV volumes and function in the setting of pediatric obesity. We found that: 1) compared to healthy weight controls, obese/overweight children have increased RV mass index without changes in RV volumes or RV ejection fraction; 2) RV systolic function, measured by free wall longitudinal strain, is impaired by 14% in obese/overweight children 3) There is evidence of a common mechanism underlying RV and LV dysfunction in obese/overweight children, since RV longitudinal strain associates with LV longitudinal strain and RV longitudinal strain is most severely impaired (by 30%) in obese children with LV concentric hypertrophy; 4) RV mass and RV longitudinal strain are related to measures of body composition; 5) inter-observer reproducibility is good for RV longitudinal strain measured with DENSE at end-systole. To our knowledge, this is the first study to comprehensively characterize RV geometry and function in obese children using CMR.

### RV remodeling and function in pediatric obesity

While RV remodeling (increased mass and volumes) and impaired systolic and diastolic function have been documented in overweight and obese adults, this information is mostly missing and inconclusive in childhood obesity. One study by Labombarda et al. [18] reported enlarged RV end-diastolic dimension in obese children compared to healthy controls, while Mahfouz et al. [19] and Zeybek et al. [20] observed no change in RV dimensions. RV systolic function in obese children was documented by a few studies using tricuspid annular plane systolic excursion (TAPSE) or systolic velocity; however, both no change [4, 18] and decreased function [19, 21] were reported. Only one study [20] quantified RV ejection fraction, a commonly used metric for ventricular function clinically, and found no significant changes in obese children. In the current study, we found increased RV mass index and comparable RV volumes and ejection fraction between obese children and healthy weight controls, suggesting that the RV remodels with preserved ventricular volumes and global systolic function in the early stages of obesity.

Measures of cardiac mechanics, such as strain, are more sensitive metrics of cardiac function than ejection fraction and are more closely related with outcomes such as death [37]. RV free wall strain and strain rate in obese children were documented by several studies using echocardiography, but their findings are again contradictory: Barbosa et al. [4] found increased RV strain and strain rate, while Di Salvo et al. [5] found decreased RV strain and strain rate in obese children. Using the advanced and highly reproducible DENSE CMR technique, our results show a 14% reduction in RV free wall longitudinal strain (Fig. 2, Table 2) in obese/overweight children compared to healthy weight controls. This finding suggests that subclinical RV contractile dysfunction exists in obese/overweight children, despite preserved RV ejection fraction.

It is worth noting that not all obese/overweight children had impaired longitudinal strain. Using previously defined cutoff values of LVMI and LV mass/volume ratio [6], we identified a subgroup of children (*n* = 10, 14%) with LV concentric hypertrophy. These children had the most impaired RV longitudinal strain (by 30%) compared to those with normal LV geometry (Fig. 4), while children with LV concentric remodeling/eccentric hypertrophy had relatively normal RV longitudinal strain. Similar findings about LV longitudinal and circumferential strain have also been reported in obese children [6]. This group of children with LV concentric hypertrophy and reduced LV and RV strain may represent a particularly high-risk phenotype with increased risk of cardiovascular disease and premature death, which deserves further investigation.

### Mechanisms for RV remodeling and dysfunction

While this study did not explore the mechanisms underlying the observed ventricular remodeling and dysfunction, which are presently not well understood, the observation of several associations of RV function with LV structure and function are suggestive of common, intrinsic causal factors. For example, insulin resistance is a common comorbidity in childhood obesity and is present in up to 50% of obese children [38]. Hyperinsulinemia in the setting of insulin resistance is a mediator of cardiac growth which could potentially result in hypertrophy and remodeling in both ventricles [39]. Further, hyperinsulinemia could alter contractile function directly [40]. Additionally, we also showed a significant correlation between RV longitudinal strain, RV mass index and measures of body composition independent of somatic growth (Table 3, Fig. 6), suggesting potential impact of excess adipose tissue on RV systolic dysfunction. This contribution of adiposity may be mediated through obesity-related metabolic changes such as insulin resistance and systemic inflammation, which could contribute to RV dysfunction through various growth factors and inflammatory markers [41, 42].

Additional explanations for the observed RV dysfunction include direct mechanical factors. Interactions between the LV and RV [43, 44] are indicated by the facts that obese/overweight children with concentric hypertrophy had the most impaired RV longitudinal strain (Fig. 4), and that LV longitudinal strain and systolic blood pressure were both correlated to RV longitudinal strain (Fig. 3). Pulmonary hypertension and obstructive sleep apnea have a significant effect on RV dysfunction; however, we did not include children who were known to have those conditions. In addition, previous studies have shown no changes in pulmonary artery pressure in obese children [5, 18].

### Imaging techniques for the RV

Due to its complex geometry and thin myocardial wall, imaging the RV is inherently difficult. All previous studies of RV function in the setting of obesity have used echocardiographic imaging techniques (Tissue Doppler or 2D speckle tracking), which suffer from poor image quality, limited acoustic windows and angle dependency. Physical interference of excessive adiposity with the imaging signal makes echocardiography even more challenging in obese subjects. CMR overcomes these limitations and is therefore superior to echocardiography for studying both geometry and function (strain) of the RV. As shown in the current study, 68% of subjects had adequate image quality for strain analysis, and the inter-observer reproducibility for RV longitudinal strain by DENSE was good with a coefficient of variation of 10%. To our knowledge, this is the first study to assess and report good reproducibility of RV longitudinal strain in obese children. The superior reproducibility of CMR is therefore favorable for detecting subclinical RV changes in obese children.

### Limitations

This study has several limitations. Cross-sectional design of the study precludes determination of onset and development of RV dysfunction in obese children. There was a small difference in age between the healthy weight and obese/overweight groups; however, this difference was accounted for during statistical analysis.

Differences in LV and RV strains between the healthy and obese/overweight groups were relatively small. Despite reasonable inter-observer reproducibility, the limits of agreement were larger than the detected difference between the groups. This could be explained by the fact that we only enrolled uncomplicated obese children in the current study, who are likely to be at the early stage of obesity. Furthermore, due to the limitation of the CMR bore circumference, most severely obese children, who were expected to have the most structural and functional impairment, were excluded from the study.

In addition, 30% of the enrolled subjects were excluded from RV strain analysis due to the poor quality of DENSE images, although demographics of these subjects were similar to those included in the analysis. Potential explanations include inconsistent breathing pattern or subject movement during the scan, which lead to blurry images and mis-registration due to through-plane motion. Future studies with higher resolution and three-dimensional DENSE imaging could improve image quality and therefore, increase the success rate in imaging the RV [24, 45].

Finally, although obesity-related metabolic changes (insulin resistance, systemic inflammation) have been shown to contribute to ventricular remodeling and dysfunction, we did not collect any blood samples to explore the role of insulin resistance and inflammatory biomarkers in RV remodeling and dysfunction observed in the current study. Future studies need to measure insulin resistance and other inflammation markers to elucidate their contribution to RV remodeling and dysfunction in obese children.

## Conclusion

Obese/overweight children have increased right ventricular mass and impaired RV contractile function compared to healthy weight children. These changes are associated with left ventricular remodeling, as those children with left ventricular concentric hypertrophy have the most impaired right ventricular longitudinal strain. These results suggest there may be a common mechanism underlying both remodeling and dysfunction of the left and right ventricles in obese/overweight children. Further study is warranted to identify these mechanisms, as well as the prognostic consequences and potential treatment options for these patients with sub-clinical cardiac dysfunction.

## Additional file

Additional file 1: Table S1. Comparison of demographic parameters between included and excluded subjects. (DOCX 51 kb)

## Abbreviations

ANCOVA: Analysis of covariance
ANOVA: Analysis of variance
BMI: Body mass index
CMR: Cardiac magnetic resonance
CoV: Coefficient of variation
DENSE: Displacement encoding with stimulated echoes
EDV: End-diastolic volume
ESV: End-systolic volume
LV: Left ventricle
LVMI: Left ventricular mass index
RV: Right ventricle
SD: Standard deviation
SSFP: Steady-state free-precession
SV: Stroke volume
