# Supplementary material for: Impaired right ventricular contractile function in childhood obesity and its association with right and left ventricular changes: a cine DENSE cardiac magnetic resonance study
Source: J Cardiovasc Magn Reson. 2017 Jun 28;19:49. doi: 10.1186/s12968-017-0363-5 (PMC5490166; doi:10.1186/s12968-017-0363-5)
Supplement: Additional file 1: Table S1. — Comparison of demographic parameters between included and excluded subjects. (DOCX 51 kb) [file 12968_2017_363_MOESM1_ESM.docx]

Table S1: Comparison of Demographic Parameters between Excluded and Included Subjects

|  | **Exclusion**  **n = 31** | **Inclusion**  **n = 70** | ***p*** |
| --- | --- | --- | --- |
| *Age (years)* | 13.8 ± 2.2 | 12.9 ± 2.8 | 0.07 |
| *Female (%)* | 45 | 51 | 0.67 |
| *Weight (kg)* | 68 ± 20 | 60 ± 22 | 0.08 |
| *Height (cm)* | 162 ± 12 | 158 ± 15 | 0.10 |
| *Body Mass Index (kg/m^2^)* | 26 ± 7 | 24 ± 7 | 0.18 |
| *Body Mass Index Percentile* | 79 ± 26 | 71 ± 30 | 0.22 |
| *Body Mass Index z-score* | 1.2 ± 1.1 | 1.0 ± 1.2 | 0.31 |
| *Obese/Overweight (%)* | 60 | 49 | 0.40 |
